# Thoracic and respirable particle definitions for human health risk assessment

**DOI:** 10.1186/1743-8977-10-12

**Published:** 2013-04-10

**Authors:** James S Brown, Terry Gordon, Owen Price, Bahman Asgharian

**Affiliations:** 1 National Center for Environmental Assessment, U.S. Environmental Protection Agency, MD B243-01, Research Triangle Park, Raleigh, NC 27711, USA; 2 NYU School of Medicine, 57 Old Forge Road, Tuxedo, NY 10987, USA; 3 Applied Research Associates, Inc, 801 N. Quincy St., Suite 700, Arlington, VA 22203, USA; 4 Applied Research Associates, Inc, 8537 Six Forks Road., Suite 600, Raleigh, NC 27615, USA

**Keywords:** Size-selective sampling, Fine and coarse particles

## Abstract

**Background:**

Particle size-selective sampling refers to the collection of particles of varying sizes that potentially reach and adversely affect specific regions of the respiratory tract. Thoracic and respirable fractions are defined as the fraction of inhaled particles capable of passing beyond the larynx and ciliated airways, respectively, during inhalation. In an attempt to afford greater protection to exposed individuals, current size-selective sampling criteria overestimate the population means of particle penetration into regions of the lower respiratory tract. The purpose of our analyses was to provide estimates of the thoracic and respirable fractions for adults and children during typical activities with both nasal and oral inhalation, that may be used in the design of experimental studies and interpretation of health effects evidence.

**Methods:**

We estimated the fraction of inhaled particles (0.5-20 μm aerodynamic diameter) penetrating beyond the larynx (based on experimental data) and ciliated airways (based on a mathematical model) for an adult male, adult female, and a 10 yr old child during typical daily activities and breathing patterns.

**Results:**

Our estimates show less penetration of coarse particulate matter into the thoracic and gas exchange regions of the respiratory tract than current size-selective criteria. Of the parameters we evaluated, particle penetration into the lower respiratory tract was most dependent on route of breathing. For typical activity levels and breathing habits, we estimated a 50% cut-size for the thoracic fraction at an aerodynamic diameter of around 3 μm in adults and 5 μm in children, whereas current ambient and occupational criteria suggest a 50% cut-size of 10 μm.

**Conclusions:**

By design, current size-selective sample criteria overestimate the mass of particles generally expected to penetrate into the lower respiratory tract to provide protection for individuals who may breathe orally. We provide estimates of thoracic and respirable fractions for a variety of breathing habits and activities that may benefit the design of experimental studies and interpretation of particle size-specific health effects.

## Background

It has long been recognized that the regional pattern of particle deposition in the respiratory tract affects the pathogenic potential of inhaled aerosols. For example, Morgan [1] concluded that respirable dusts likely caused pneumoconiosis and silicosis in coal miners, whereas a larger size fraction caused bronchitis and obstructive changes in pulmonary function. Sampling the total air concentration of particulate matter (PM) provides a crude estimate of exposure that may not correlate with observed health effects if the risk is associated only with those particles that may enter the thorax or penetrate beyond the ciliated airways. The concept of size-selective particle sampling has been employed as a means for effectively sampling the particle sizes associated with specific pathologic outcomes (e.g., the respirable fraction with parenchymal disease). If an environmentally or occupationally related particle is recognized to only affect the gas-exchange region of the lung, then a sampling strategy that only collects the respirable fraction of airborne PM is preferable to sampling total suspended particulate (TSP) or the thoracic fraction.

The human respiratory tract can be divided into three main regions based on size, structure, and function, namely, the head, tracheobronchial region (also known as the conducting airways), and the gas-exchange region (also known as the parenchymal, alveolar, or pulmonary) region. Size-selective sampling is intended to help discern the amount of aerosol expected to be available for deposition in a region. Most sampling conventions have been defined in terms of particle penetration into respiratory regions rather than the expected particle deposition or dose to regions. Specific definitions used herein, adopted from the European Committee for Standardization (CEN), are [2]:

•Inhalable fraction – the mass fraction of total airborne particles which is inhaled through the nose and mouth.

•Extrathoracic fraction – the mass fraction of inhaled particles failing to penetrate beyond the larynx.

•Thoracic fraction – the mass fraction of inhaled particles penetrating beyond the larynx.

•Respirable fraction – the mass fraction of inhaled particles penetrating to the unciliated airways.^a^

The above definitions are stated in terms of a mass fraction. Relative to total airborne particles, the particle size having 50% penetration for the thoracic and respirable fractions are 10 μm and 4.0 μm (all particle sizes are aerodynamic diameter unless expressed otherwise), respectively [2,3]. These criteria were specifically developed for workplace atmospheres. Since particles must generally become deposited to exert biological effects, these conventions, based on regional exposure (i.e., particles penetrating into a region of the respiratory tract), are conservative by design in that they overestimate the amount of inhaled material that becomes deposited and thereby available to induce an effect.

**Figure 1:**
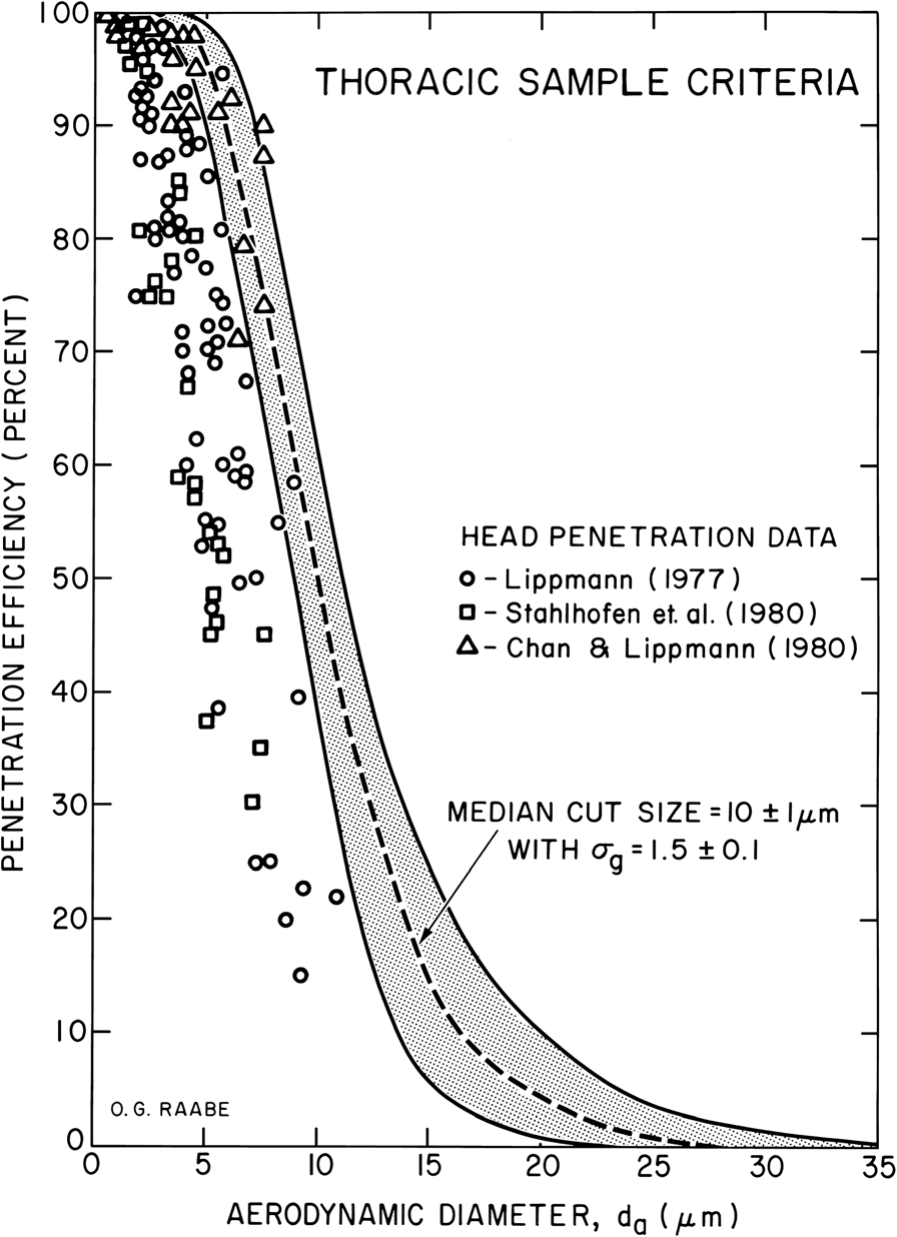
**Thoracic particulate mass fraction criteria (relative to total airborne particles) for size-selective sampling.** Individual data points are observed human head penetration efficiency during oral inhalation for an inspiratory flow rate of 43.5 L/min, i.e., light exercise [5-7]. As stated by ACGIH [3], the sampling criterion is offset to the right of experimental data to overestimate the amount of exposure to the lower respiratory tract, i.e., the lungs, and correspondingly to provide a greater level of protection for exposed workers. From ACGIH^®^, *Particle Size-Selective Sampling in the Workplace, Report of the ACGIH^®^ Technical Committee on Air Sampling Procedures*. Copyright 1985. Reprinted with permission. Courtesy: Dr. Otto G. Raabe.

In 1985, the American Conference of Governmental Industrial Hygienists (ACGIH) recommended particle size-selective sampling in setting threshold limit values for occupational exposures [4].^b^ The ACGIH specifically considered a reference worker (weight, 70 kg; height, 175 cm) breathing orally while engaged in light activity (minute ventilation, 21.75 liters/min). Criteria were established for Inspirable (now Inhalable), Thoracic, and Respirable Particulate Mass that were intended to be protective against materials that were considered hazardous when deposited anywhere in the respiratory tract, anywhere within the lungs, and in the gas-exchange region, respectively. These criteria were based on exposure of a respiratory tract region (based on particle penetration into that region), not particle deposition in a respiratory tract region. The ACGIH committee recognized uncertainty related to individual biological variability in respiratory health status, breathing patterns (rate and route), and airways structure as well as differences in work rates, all of which can cause differences in inhaled aerosol deposition and dose. Facing these uncertainties, the committee afforded extra protection to exposed workers by over representing the true penetration of particles into regions of the respiratory tract as illustrated in Figure 1[4].

Size-selective sampling has also been employed by the U.S. Environmental Protection Agency (EPA) in setting the national ambient air quality standards (NAAQS) for particulate matter (PM). In 1987, the EPA changed the indicator for PM from TSP (effectively an aerodynamic cut-size varying from 25 to 40 μm, depending on wind speed and direction) to PM_10_ (particles with a nominal mean aerodynamic diameter ≤ 10 μm) [8]. Consistent in concept with the ACGIH thoracic particle fraction, PM_10_ delineates a subset of inhalable particles (referred to as thoracic particles) that are thought small enough to penetrate to the thoracic region (including the tracheobronchial and alveolar regions) of the respiratory tract.^c^ In 1997, the EPA extended size-selective sampling to include fine particles indicated by PM_2.5_ (particles with a nominal mean aerodynamic diameter ≤ 2.5 μm) and retained PM_10_ as the indicator for thoracic coarse particles [9]. The selection of PM_2.5_ by the EPA was mainly to delineate the atmospheric fine (combustion derived, aggregates, acid condensates, secondary aerosols) and coarse (crustal, soil-derived dusts) PM modes and for consistency with community epidemiologic health studies reporting various health effects associated with PM_2.5_. With consideration to the PM NAAQS, Miller et al. [10] also specifically recommended a particle size cut-point of ≤ 2.5 μm as an indicator for fine PM based on consideration of particle penetration into the gas-exchange region and the delineation of the fine and coarse particle modes.

Most recently, the International Organization for Standardization (ISO) has released recommendations for sampling conventions based on particle deposition (rather than exposure) in adult males and females engaged in activities of sitting, light exercise and heavy exercise as specified in Table 1[11]. The ISO estimates of deposition were determined using the International Commission on Radiological Protection (ICRP) human respiratory tract model [12]. These new ISO conventions [11] are not considered further herein as current sampling conventions for occupational and non-occupational settings remain dependent on the probability of particle penetration rather than deposition in specific regions of the respiratory tract.

**Table 1:** Ventilatory and activity patterns for adult males, adult females, and a ten year-old child

		**Sleeping**	**Sitting**	**Light**	**Heavy**
				**Exercise**	**Exercise**
Adult Male	V_T_ (mL)	625	750	1250	1920
Sedentary worker	f (min^-1^)	12	12	20	26
	t (hr)	8.5	5.5	9.75	0.25
	V_daily_ (L/day)	3825	2970	14625	749
Adult Female	V_T_ (mL)	444	464	992	1364
Sedentary worker	f (min^-1^)	12	14	21	33
	t (hr)	8.5	5.5	9.75	0.25
	V_daily_ (L/day)	2717	2144	12187	675
Child (10 yrs)	V_T_ (mL)	304	333	583	752
Male or Female	f (min^-1^)	17	19	32	45
	t (hr)	10	4.67	9.33	0
	V_daily_ (L/day)	3101	1772	10447	0

V_T_, tidal volume; f, breathing frequency; t, time spent engaged in specific activity; V_daily_, total volume inspired in 24 hr. Data are from ICRP [12] Tables B15 and B16A-B for breathing and activity patterns, respectively.

Conceptually, size-selective sampling better characterizes PM exposure to regions of the respiratory tract and thereby affords more appropriate avenues for protection of exposed populations than TSP. Such a simple concept is not, however, without ambiguity in definitions and debate over appropriate sampling approaches. For example, the definition for the thoracic fraction specifies particles “penetrating beyond the larynx,” whereas the ACGIH thoracic convention for sampling (Figure 1) clearly and intentionally overestimates the fraction of large particles penetrating into the thoracic region to afford extra protection of occupationally exposed individuals. The purpose of this paper is to provide realistic estimates of thoracic and respirable particle fractions for adults and children that may be used in the design of experimental studies and interpretation of health effects evidence.

The ICRP human respiratory tract model [12] was used to estimate particle penetration through the extrathoracic (ET) airways. The ICRP predictive equations for ET deposition are based on experimental measurements in humans. Although also based on human data, the ICRP model was not used to estimate penetration through the tracheobronchial (TB) airways due to its reliance on measurements of particle clearance from the TB airways and the ability to target particle deposition into the ciliated airways. That is, much of the available regional deposition data for the TB and alveolar regions have been obtained from experiments with radioactively labeled, poorly soluble particles or by use of aerosol bolus techniques (see Sections D.9.2 and E.5.3 of Ref [12]). Aerosol bolus (40 ml volume of 3.5 μm particles) inhaled to a very shallow lung volume (70 ml, ~75% of phase I inert gas washout) by healthy adults (10 M, 6 F; 20-43 yrs of age) show preferential left lung deposition and 23% retention at 48 hrs [13]. This suggests slow TB airway clearance and/or some penetration into the alveolar region. Given the above, coupled with uncertainty related to slow TB clearance [e.g., [14,15], we utilized the publicly available multiple path particle dosimetry (MPPD; ver 2.1, © 2009) model to estimate penetration through the TB airways.

## Methods

Once particles have entered the respiratory tract via the nose or mouth, the primary factors affecting particle penetration into the lower respiratory tract (i.e., beyond the larynx) are airways size and structure, breathing pattern (flow and volume), route of breathing (nose vs. mouth), and inhaled particle size. With regard to particle size, we have considered particles whose deposition is governed by their inertial properties, i.e., ≥ 0.5 μm. Breathing patterns vary mainly by sex, age, and activity. Table 1 provides the breathing patterns, subject groups, and activity patterns from the ICRP [12] model that were used in our assessment.

Based on our comparison of the ICRP model [12] to more recent data provided by Brochu et al. [16], the daily ventilation rates and activity patterns provided in Table 1 overestimate typical daily ventilation rates. Table 2 provides daily ventilation rates (5^th^, 50^th^, and 95^th^ percentiles) reported by Brochu et al. [16]. The daily ventilation rates from the ICRP [12] model’s recommended time budget roughly correspond to the highly active 95^th^ percentile (see Table 1 vs. Table 2). To assess the effect of daily activity/ventilation rates on particle penetration into the respiratory tract, we reduced the estimated time (given in Table 1) that individuals spent engaged in light exercise and correspondingly increased their time sitting to match the daily ventilation rates in Table 2. To match the very sedentary 5^th^ percentile of daily activity, it was necessary also to eliminate time spent by the adult female engaged in heavy exercise and to attribute all of the child’s activity to sleeping. Even when considering 100% of the child’s activity equivalent to sleeping, the ICRP [12] breathing pattern slightly overestimated (by 3%; 7.4 vs. 7.2 m^3^/day) the 5^th^ percentile daily ventilation rates of Brochu et al. [16].

**Table 2:** **Daily ventilation rates (V**_**daily**_**).**^**a**^

	**V**_**daily **_**(m**^**3**^**/day)**		
	**Very sedentary**	**Median**	**Highly active**
	**5**^**th **^**percentile**	**50**^**th **^**percentile**	**95**^**th **^**percentile**
Male ^b^	12.86	17.48	22.11
Female ^b^	9.91	13.67	17.42
Children ^c^	7.20	10.22	13.24

^a^ Data are from Table 2 of Brochu et al. [16]; ^b^ values for age range of 23 to <30 years; ^c^ average of male and female values for age range of 7 to <11 years.

Route of breathing varies with inspiratory flows and generally shifts from nasal to oronasal breathing at higher flows. The ICRP [12] model characterizes breathing habit based on Niinimaa et al. [17], who examined the route of breathing as a function of activity in healthy adults (14 males, 16 females). Eighty-seven percent of the subjects breathed through the nose at rest and switched to oronasal breathing with exercise. These subjects were referred to as “*normal augmenters*.” Thirteen percent breathed oronasally even at rest and were referred to as “*mouth-breathers*.” For both of these breathing habits (i.e., normal augmenters and mouth-breathers, we estimated the fraction of a breath passing through the oral and nasal pathways from regression equations for oral breathing in Figure 1 of the Niinimaa et al. study [17]. The ICRP [12] model utilizes this same general approach, but for each breathing habit and activity (i.e., sleeping, sitting, etc.), the same fraction of oral breathing was assumed applicable to all ages and both sexes.

In considering breathing habit, we differed from the ICRP, in that we assumed the fraction of oral breathing to differ between adult males and females as a function of their minute ventilation rather than their activity level. However, children tend to have a greater fraction of oral breathing than adults at rest and during exercise [18,19]. Therefore, consistent with the ICRP, we assumed the fraction of the breath inhaled through the mouth (F_m_) in the child engaged in some specific level of activity was equal to that of the adult male engaged in the same level of activity despite the dramatically lower ventilation rates of the child. We also considered recent breathing habit data not available for inclusion in the ICRP model [12]. Bennett et al. [19,20] show a more gradual increase in oronasal breathing than did Niinimaa et al. [12]. In addition to the normal augmenter and mouth-breather breathing habits based on the Niinimaa et al. [17] study, we also considered the more gradual onset of oronasal breathing observed in adults and children by the Bennett et al. [19,20] studies, herein termed as “*gradual augmenters*.”

The gradual augmenter breathing habit for children was estimated by linear regression of the observed minute ventilation and F_m_ at rest and at 40% maximum physical work capacity from data in Table two and Figure two of Bennett et al. [19] for 12 children (9 M, 3 F; 6-10 yrs of age). The gradual augmenter breathing habit for adult males and females was estimated by linear regression of the observed minute ventilation and F_m_ at rest and at 60% maximum physical work capacity from data in Table two and Figure three of Bennett et al. [20] for 22 adults (11 M, 11 F; mean age, 22 yrs). In the adult females, the fitted F_m_ was zero for the activity of sitting and so was also set to zero for the activity of sleep. Table 3 provides the F_m_ for all breathing habits (normal augmenters, mouth-breathers, and gradual augmenters) used in our simulations. In a study of 37 subjects from 7-72 years of age, James et al. [21] reported that 2 subjects (5.4%) breathed orally only. With this finding in mind, we have also considered purely oral breathing in our estimates of particle penetration into regions of the lower respiratory tract.

**Table 3:** Partitioning of breaths through the mouth and nose

		**Sleeping**	**Sitting**	**Light**	**Heavy**
				**Exercise**	**Exercise**
Adult Male	Normal Augmenter ^a^	0, 1.00 ^d^	0, 1.00	0, 1.00	0.52, 0.48
	Mouth-breather ^a^	0.29, 0.71	0.36, 0.64	0.59, 0.41	0.66, 0.34
	Gradual Augmenter ^b^	0.12, 0.88	0.13, 0.87	0.29, .071	0.54, 0.46
Adult Female	Normal Augmenter ^a^	0, 1.00	0, 1.00	0, 1.00	0.50, 0.50
	Mouth-breather ^a^	0.12, 0.88	0.23, 0.77	0.57, 0.43	0.65, 0.35
	Gradual Augmenter ^b^	0, 1.00	0, 1.00	0.22, 0.78	0.59, 0.41
Child	Normal Augmenter ^a^	0, 1.00	0, 1.00	0, 1.00	0.51, 0.49
	Mouth-breather ^a^	0.29, 0.71	0.36, 0.64	0.59, 0.41	0.66, 0.34
	Gradual Augmenter ^c^	0.29, 0.71	0.31, 0.69	0.51, 0.49	0.77, 0.23

^a^ From regression equations for oral breathing in Figure 1 of Niinimaa et al. [17]; ^b^ Data based on Table 2 and Figure 3 of Bennett et al. [20] for 22 individuals (11 M, 11 F; mean age, 22 yrs); ^c^ Data based on Table 2 and Figure 2 of Bennett et al. [19] for 12 children (9 M, 3 F; 6-10 yrs of age); ^d^ fraction inhaled through mouth, fraction inhaled through nose.

For air passing through the mouth, deposition of large particles by impaction occurs mainly at the larynx. From Eq D.30 of ICRP [12], laryngeal deposition efficiency, η(ET)_larynx_, is given by:

(1) $$\eta {\left(\mathrm{ET}\right)}_{\mathrm{larynx}}=1-{\left\{1.1\times 10^{-4}{\left[\;d_{a}^{2}\;{\left(Q_{\mathrm{total}}\;SF^{3}\right)}^{0.6}\;{\left(\mathrm{Vt}\;SF_{t}{}^{3}\right)}^{-0.2}\;\right]}^{\;1.4}\;+\;1\right\}}^{-1}\;$$

where: d_a_ is aerodynamic diameter (μm); Q_total_ is total inspiratory flow rate (mL/s); V_T_ is tidal volume (mL); and SF_t_ is a scaling factor of 1.0 for adult males, 1.08 for adult females, and 1.26 for ten year-old children from Table fifteen of ICRP [12].

For nasal breathing, ET deposition efficiency due to impaction was calculated from Eq. D.32 and D.33 of ICRP [12]. The ET deposition efficiencies for the anterior, η(ET_1_)_nose_, and posterior, η(ET_2_)_nose_, nasal regions are given by:

(2) $$\eta {\left(ET_{1}\right)}_{\mathrm{nose}}=0.5\;\left\{\;1-{\left[3\times 10^{-4}\;\left(d_{a}^{2}\;Q_{\mathrm{nose}}\;SF_{t}{}^{3}\right)+1\;\right]}^{-1}\right\}\;$$

(3) $$\eta {\left(ET_{2}\right)}_{\mathrm{nose}}=1-\;{\left[\;5.5\times 10^{-5}\;{\left(d_{a}^{2}\;Q_{\mathrm{nose}}\;SF_{t}{}^{3}\right)}^{1.17}+1\;\right]}^{-1}\;$$

where: Q_nose_ is the inspiratory flow (mL/s) through the nose. The use of SF_t_ in Equations 2 and 3 presumes that nasal deposition efficiency increases with decreasing body size and increasing nasal resistance. Two studies [19,22] suggest that the nasal deposition in children is less than that of adults. These two studies, not considered in the ICRP model [12], suggest that it may be inappropriate to apply a scaling factor for nasal deposition of children. Accordingly, we estimated the nasal deposition efficiency of the 10 yr old child for a SF_t_ of both 1.0 (child-A) and 1.26 (child-B). Additionally, we estimated the upper and lower 95% confidence bounds for inter-individual variability attributable to differences in deposition efficiency within the ET region predicted by Equations 1-3 as specified in paragraphs D44 and D68 of ICRP [12].

The deposition efficiencies along the ET pathways (i.e., nasal and oral) were assumed to be independent. As such, total ET deposition was taken to be the sum of deposition between the pathways weighted by the flow partitioning (see Paragraph 161 of ICRP [12]). The thoracic fraction, defined as particle penetration past the larynx, P(ET), is given by:

(4) $$\begin{array}{l} P\left(\mathrm{ET}\right)=1-\;F_{m}\;\eta {\left(\mathrm{ET}\right)}_{\mathrm{larynx}}-\left(1-F_{m}\right)\\ \;\times \left[\;\eta {\left(ET_{1}\right)}_{\mathrm{nose}}+\;\left(\;1-\;\eta {\left(ET_{1}\right)}_{\mathrm{nose}}\right)\;\eta {\left(ET_{2}\right)}_{\mathrm{nose}}\;\right]\; \end{array}$$

We estimated inspiratory deposition efficiency in the TB region, η_TB_, of particles (0.5-20 μm; 0.1 μm increments) using the publicly available multiple path particle dosimetry (MPPD; ver 2.1, © 2009) model.^d^ The model considers deposition by the mechanisms of impaction, sedimentation, and diffusion. The approach and formula used to calculate particle losses in the MPPD model are described by Anjilvel and Asgharian [23]. Physiological input parameters (namely, tidal volume [V_T_], breathing frequency [f], functional residual capacity [FRC], and upper respiratory tract volume [URT]), necessary for MPPD simulations are provided in Tables 1 and 4. FRC and URT for each group are from Table fifteen of ICRP [12]. The Yeh and Schum [24] typical path whole lung model was utilized and scaled for FRC and V_T_. The effects of these physiologic parameters on deposition in humans free of respiratory disease are described by de Winter-Sorkina and Cassee [25].

**Table 4:** Functional residual volume (FRC) and upper respiratory tract volumes (URT)

	**FRC (mL)**	**URT (mL)**
Adult Male	3300	50
Adult Female	2680	40
Child (10 yrs)	1484	25

**Figure 2:**
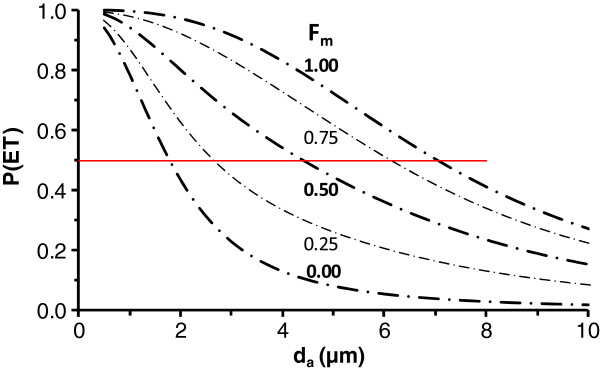
**Thoracic fraction, i.e., particle penetration through the extrathoracic region, P(ET), as a function of breathing route.** Penetration data are with respect to particle diameter as a function of the fraction of air inhaled through the mouth (F_m_) in an adult male engaged in light exercise relative to particles entering the respiratory tract. Curves are for the F_m_ of 0.00, 0.25, 0.50, 0.75, and 1.00 as indicated on the figure. Horizontal red line highlights 50% penetration.

**Figure 3:**
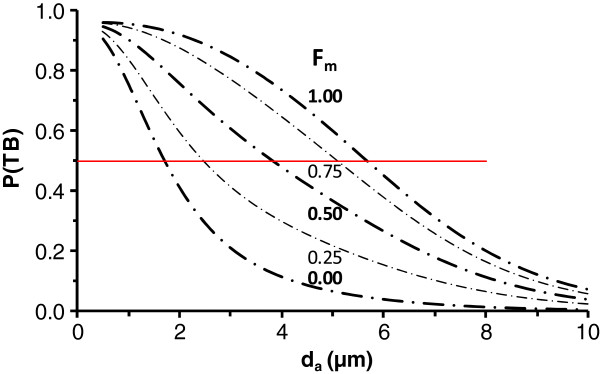
**Respirable fraction, i.e., particle penetration through the tracheobronchial region, P(TB), as a function of breathing route.** Penetration data are with respect to particle diameter as a function of the fraction of air inhaled through the mouth (F_m_) in an adult male engaged in light exercise relative to particles entering the respiratory tract. Curves are for the F_m_ of 0.00, 0.25, 0.50, 0.75, and 1.00 as indicated on the figure. Horizontal red line highlights 50% penetration.

The respiratory fraction, defined as particle penetration through the ciliated airways of the TB region, P(TB), is given by:

(5) $$P\left(\mathrm{TB}\right)=\frac{\int_{\;0}^{\;T_{\mathrm{inh}}}C_{1}\;\mathrm{dt}}{\int_{\;0}^{\;T_{\mathrm{inh}}}C_{0}\;\mathrm{dt}}≅P\left(\mathrm{ET}\right)\;\left(\;1-\eta_{\mathrm{TB}}\;\right)\;$$

where: C_0_ and C_1_ are particle concentration passing the larynx and terminal bronchioles, respectively; and T_inh_ is the time of inhalation. Since conducting airway particle concentration is nearly constant during inhalation, respiratory fraction can be expressed in terms of TB deposition efficiency as given above. An Additional file 1: Appendix to this paper provides estimates of P(TB) based on the ICRP [12] model rather than the MPPD model.

After calculating P(ET) and P(TB) for all activities and individual groups, daily average estimates of P(ET)_avg_ and P(TB)_avg_ weighted by daily ventilation (see Tables 1 and 2) were calculated as a function of particle size. Ventilation-weighted averages of P(ET)_avg_ for each particle size were calculated as:

(6) $$P{\left(\mathrm{ET}\right)}_{\mathrm{avg}}\;=\sum_{i=1}^{n}\left[P{\left(\mathrm{ET}\right)}_{i}\;V_{\mathrm{daily}-i}\right]/\sum_{i=1}^{n}V_{\mathrm{daily}-i}\;$$

where: P(ET)_i_ is the ET fraction for activity, *i*; V_daily-i_ is the daily volume inhaled while engaged in activity, *i*; and *n* is the number of activities. Ventilation-weighted averages of P(TB)_avg_ were computed similarly to those of P(ET)_avg_.

Finally, thoracic and respiratory particle fractions were also calculated after applying the ICRP [12] inhalability criterion assuming no ambient wind, 1−0.5 [1− (0.00076 d_a_^2.8^ + 1) ^−1^]. The ICRP [12] criterion was utilized as it better represents the inhalation of particles <10 μm than the ACGIH and CEN [2,3] criterion.

## Results

We estimated particle penetration fractions into the thorax and respiratory region of an adult male, adult female, and a 10 yr old child. In the results described below, data do not consider particle inhalability unless specifically stated. Inhalability was not considered since, as the results will show, it has a minimal affect on 50% cut-points for particle penetration into the lower airways for all breathing habits except the less probable condition of oral breathing.

### Route of breathing

Of the factors we considered, route of breathing (or breathing habit) had the greatest effect on estimates of P(ET) and P(TB). Figure 2 and 3 illustrate P(ET) and P(TB) for an adult male engaged in light exercise as a function of F_m_. In Figure 2, fifty percent P(ET) occurs at the d_a_ of 1.8 , 2.7, 4.4, 6.1, and 7.0 μm for the F_m_ of 0.00, 0.25, 0.50, 0.75, and 1.00, respectively. Note that the curve in Figure 2 for F_m_=1 is for comparable conditions to those for which ACGIH [4] thoracic fraction was based, i.e. an orally breathing adult male engaged in light exercise. In Figure 3, fifty percent P(TB) occurs at the d_a_ of 1.7 , 2.5, 3.8, 5.1, and 5.7 μm for the F_m_ of 0.00, 0.25, 0.50, 0.75, and 1.00, respectively. As ventilation is shifted to the lower removal efficiency oral passages, there is an ever greater separation between the P(ET) and P(TB) curves. By contrast, for purely nasal breathing (F_m_ = 0, the case for normal augmenters during light exercise), due to the vast removal of particles in the nasal airways, there is nearly no difference between the P(ET) and P(TB) curves in Figures 2 and 3, respectively.

Table 5 provides the 50% cut-points for particle penetration into the thorax and respiratory region for all the breathing habits we evaluated. As may be expected based on F_m_ (see Table 3), the predicted particle penetration for the gradual augmenter breathing habit is enveloped between that of the normal augmenter and mouth-breathers. Additionally, consistent with Figures 2 and 3, Table 5 shows that the largest 50% cut-points are observed during the case of oral breathing. Table 6 provides data on the penetration of 10 μm particles into the lower respiratory tract which is generally less than 20%, except for the case of oral breathing where penetration into the thorax can approach 40%.

**Table 5:** Particle penetration (50% cut-point) through respiratory tract regions relative to particles entering the respiratory tract

**V**_**daily **_**(%-tile)**	**Normal**				**Gradual**			
	**Augmenter**		**Mouth-breather**		**Augmenter**		**Oral only**	
	**P(ET)**_**avg**_	**P(TB)**_**avg**_	**P(ET)**_**avg**_	**P(TB)**_**avg**_	**P(ET)**_**avg**_	**P(TB)**_**avg**_	**P(ET)**_**avg**_	**P(TB)**_**avg**_
**Male**								
5%	2.94 ^a^	2.74	5.15	4.32	3.60	3.25	9.00	6.67
95% CI	(1.72–5.03)	(1.65–4.34)	(3.12–8.48)	(2.84–6.10)	(2.11–6.11)	(2.00–4.98)	(5.86–13.8)	(5.00–8.14)
50%	2.46	2.30	5.09	4.30	3.37	3.05	8.09	6.11
95% CI	(1.44–4.20)	(1.38–3.71)	(3.14–8.26)	(2.86–5.88)	(1.99–5.67)	(1.88–4.65)	(5.26–12.4)	(4.58–7.34)
95%	2.14	2.03	5.08	4.31	3.19	2.90	7.61	5.89
95% CI	(1.25–3.66)	(1.20–3.32)	(3.16–8.16)	(2.89–5.83)	(1.90–5.33)	(1.79–4.44)	(4.95–11.7)	(4.38–7.07)
**Female**								
5%	2.92	2.71	4.32	3.78	3.10	2.86	8.78	6.50
95% CI	(1.71–5.01)	(1.63–4.28)	(2.57–7.22)	(2.40–5.52)	(1.81–5.30)	(1.73–4.48)	(5.71–13.5)	(4.88–7.91)
50%	2.42	2.27	4.46	3.87	2.91	2.68	7.88	5.96
95% CI	(1.41–4.13)	(1.36–3.64)	(2.72–7.32)	(2.51–5.45)	(1.71–4.94)	(1.63–4.19)	(5.13–12.1)	(4.47–7.17)
95%	2.10	1.99	4.58	3.96	2.74	2.54	7.44	5.77
95% CI	(1.23–3.60)	(1.18–3.26)	(2.82–7.42)	(2.60–5.49)	(1.61–4.64)	(1.53–4.00)	(4.84–11.4)	(4.29–6.94)
**Child-B **^**b**^								
5%	2.77	2.61	4.31	3.78	4.30	3.78	8.34	6.46
95% CI	(1.62–4.75)	(1.56–4.18)	(2.58–7.19)	(2.40–5.57)	(2.57–7.17)	(2.40–5.56)	(5.42–12.8)	(4.74–8.04)
50%	2.25	2.13	4.37	3.81	4.07	3.59	7.50	5.81
95% CI	(1.31–3.85)	(1.27–3.48)	(2.67–7.15)	(2.47–5.35)	(2.46–6.71)	(2.29–5.15)	(4.88–11.5)	(4.32–7.08)
95%	1.89	1.81	4.34	3.76	3.98	3.49	6.91	5.33
95% CI	(1.11–3.24)	(1.07–2.98)	(2.69–7.03)	(2.48–5.11)	(2.45–6.47)	(2.27–4.88)	(4.49–10.6)	(4.01–6.36)
**Child-A **^**c**^								
5%	3.92	3.56	5.60	4.72	5.59	4.72	8.34	6.46
95% CI	(2.29–6.72)	(2.18–5.44)	(3.38–9.28)	(3.09–6.63)	(3.37–9.27)	(3.09–6.62)	(5.42–12.8)	(4.74–8.04)
50%	3.18	2.94	5.37	4.53	5.12	4.36	7.50	5.81
95% CI	(1.86–5.45)	(1.77–4.58)	(3.29–8.77)	(3.03–6.10)	(3.11–8.40)	(2.88–5.95)	(4.88–11.5)	(4.32–7.08)
95%	2.68	2.50	5.16	4.34	4.85	4.14	6.91	5.33
95% CI	(1.56–4.58)	(1.50–3.97)	(3.19–8.34)	(2.94–5.66)	(2.98–7.90)	(2.76–5.51)	(4.49–10.6)	(4.01–6.36)

P(ET)_avg_, extrathoracic particle penetration which is the thoracic particle fraction averaged across all activity levels weighted by daily ventilation; P(TB)_avg_, tracheobronchial particle penetration which is the respirable particle fraction averaged across all activity levels weighted by daily ventilation; 95% CI, ninety-five percent confidence intervals for inter-individual variability attributable to differences in particle penetration through the extrathoracic region; ^a^ Aerodynamic particle diameter in μm; ^b^ Scaling factor in Equations 2 and 3 equal to 1.26; ^c^ Scaling factor in Equations 2 and 3 equal to 1.0.

**Table 6:** **Penetration of 10 μm (d**_**a**_**) through respiratory tract regions relative to particles entering the respiratory tract**

**V**_**daily **_**(%-tile)**	**Normal**				**Gradual**			
	**Augmenter**^**a**^		**Mouth-breather**^**a**^		**Augmenter**^**a**^		**Oral only**^**a**^	
	**P(ET)**_**avg**_	**P(TB)**_**avg**_	**P(ET)**_**avg**_	**P(TB)**_**avg**_	**P(ET)**_**avg**_	**P(TB)**_**avg**_	**P(ET)**_**avg**_	**P(TB)**_**avg**_
**Male**								
5%	0.05^b^	0.03	0.21	0.10	0.11	0.05	0.43	0.21
50%	0.04	0.02	0.19	0.08	0.11	0.04	0.36	0.15
95%	0.03	0.01	0.19	0.06	0.10	0.03	0.32	0.12
**Female**								
5%	0.06	0.03	0.15	0.07	0.06	0.03	0.41	0.20
50%	0.04	0.02	0.15	0.06	0.07	0.02	0.35	0.14
95%	0.03	0.01	0.16	0.05	0.07	0.02	0.31	0.11
**Child-B **^**c**^								
5%	0.05	0.02	0.16	0.08	0.16	0.08	0.38	0.18
50%	0.03	0.01	0.15	0.06	0.14	0.06	0.31	0.13
95%	0.02	0.01	0.14	0.04	0.13	0.04	0.27	0.09
**Child-A **^**d**^								
5%	0.10	0.05	0.21	0.10	0.21	0.10	0.38	0.18
50%	0.07	0.04	0.19	0.09	0.18	0.07	0.31	0.13
95%	0.05	0.02	0.17	0.05	0.16	0.05	0.27	0.09

P(ET), extrathoracic penetration for 10 μm particles; P(TB), tracheobronchial penetration for 10 μm particles; ^a^Data are daily averages, all activity levels weighted by daily ventilation; ^b^Fraction penetration; ^c^Scaling factor in Equations 2 and 3 equal to 1.26; ^d^Scaling factor in Equations 2 and 3 equal to 1.0.

### Age and sex

**Figure 4:**
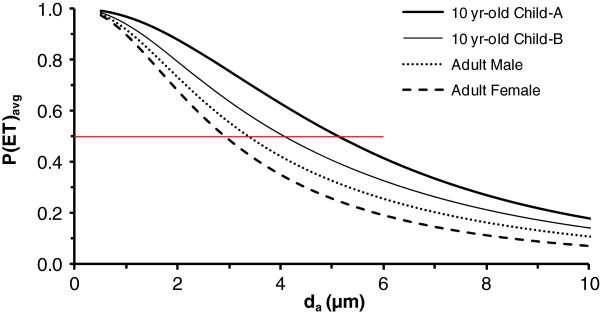
**Thoracic fraction, i.e., particle penetration through the extrathoracic region, P(ET)**_**avg**_**, in adults and a 10 yr-old child.** Data are daily averaged values for a median activity level, gradual augmenter breathing habit, and uncorrected for particle inhalability. Child-A and child-B are for a scaling factor of 1.0 and 1.26 in Equations 2 and 3, respectively. Horizontal red line highlights 50% penetration which occurs at 3.1 μm (adult female), 3.4 μm (adult male), 4.1 μm (child-B), and 5.1 μm (child-A).

**Figure 5:**
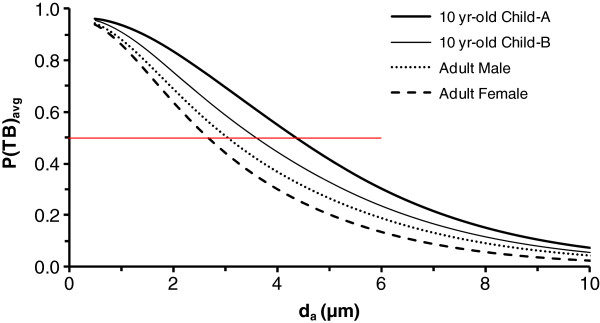
**Respirable fraction, i.e., particle penetration through the tracheobronchial region, P(TB)**_**avg**_**, in adults and a 10 yr-old child.** Data are daily averaged values for a median activity level, gradual augmenter breathing habit, and uncorrected for particle inhalability. Child-A and child-B are for a scaling factor of 1.0 and 1.26 in Equations 2 and 3, respectively. Horizontal red line highlights 50% penetration which occurs at 2.7 μm (adult female), 3.1 μm (adult male), 3.6 μm (child-B), and 4.4 μm (child-A).

Daily weighted penetrations curves for P(ET)_avg_ and P(TB)_avg_ are illustrated in Figures 4 and 5, respectively. For normal augmenters and oral breathing, 50% cut-points for P(ET)_avg_ and P(TB)_avg_ were generally similar between adult males and females, but shifted to slightly (<0.2 μm) smaller particle sizes in the females (see Table 5). There was a larger (<0.8 μm) difference in 50% cut-points between males and females for the mouth-breather and gradual augmenter breathing habits which is attributable to greater nasal inhalation by females than males.

The data for child-A are more consistent, than child-B, with experimental data [19,22] showing a lower nasal particle removal efficiency in children than adults. For all breathing habits except oral breathing in Table 5, child-A shows larger 50% cut-points than adults. Additionally, in Table 6, the penetration of 10 μm particles into the thoracic and respiratory regions of child-A is generally greater than or equal to that of adults for all breathing habits other than oral. For oral breathing, the penetration of 10 μm particles is lower in child-A than adults.

### Activity level

Impaction in the nasal airways, larynx, and large bronchi increases in conjunction with activity and increasing inspiratory flows across the range of d_a_. Therefore, decreasing 50% cut-points for both P(ET)_avg_ and P(TB)_avg_ are observed with increasing activity. In general, the penetration of 10 μm particles into the thoracic and respiratory regions also showed a small reduction with increasing daily activity level (see Table 6). However, the small reductions in particle penetration pale in comparison to the large increases in ventilation and intake of particles that occur with increasing activity level.

### Inhalability adjustment

The thoracic and respirable fraction data in Table 5 were relative to particles entering the respiratory tract. For comparison, Table 7 provides d_a_ associated with 50% penetration into the thorax and respiration regions after applying inhalability criterion. Adjusting for inhalability shifts penetration curves to smaller particle sizes, and this effect is most evident where there is a low activity level and a substantial contribution of oral breathing. Table 6 provides data on the penetration of 10 μm particles into the lower respiratory tract. Those penetration data may be adjusted for inhalability by multiplying by 0.84, the inhalability of 10 μm particles.

**Table 7:** Inhalability adjusted particle penetration (50% cut-point) through respiratory tract regions

**V**_**daily **_**(%-tile)**	**Normal**				**Gradual**			
	**Augmenter**		**Mouth-breather**		**Augmenter**		**Oral only**	
	**P(ET)**_**avg**_	**P(TB)**_**avg**_	**P(ET)**_**avg**_	**P(TB)**_**avg**_	**P(ET)**_**avg**_	**P(TB)**_**avg**_	**P(ET)**_**avg**_	**P(TB)**_**avg**_
**Male**								
5%	2.92 ^a^	2.72	4.97	4.24	3.55	3.22	8.24	6.38
95% CI	(1.71–4.90)	(1.64–4.26)	(3.09–7.66)	(2.81–5.85)	(2.11–5.82)	(2.00–4.85)	(5.66–11.4)	(4.89–7.59)
50%	2.44	2.30	4.93	4.21	3.33	3.02	7.52	5.90
95% CI	(1.43–4.12)	(1.37–3.67)	(3.11–7.51)	(2.84–5.67)	(1.99–5.43)	(1.87–4.54)	(5.12–10.5)	(4.50–6.98)
95%	2.14	2.02	4.92	4.22	3.15	2.88	7.14	5.71
95% CI	(1.25–3.62)	(1.20–3.29)	(3.13–7.44)	(2.87–5.64)	(1.89–5.15)	(1.78–4.36)	(4.84–10.1)	(4.32–6.77)
**Female**								
5%	2.90	2.70	4.22	3.72	3.08	2.85	8.06	6.23
95% CI	(1.70–4.87)	(1.63–4.20)	(2.56–6.73)	(2.38–5.34)	(1.81–5.13)	(1.73–4.40)	(5.53–11.2)	(4.78–7.41)
50%	2.41	2.26	4.36	3.81	2.89	2.67	7.35	5.77
95% CI	(1.41–4.06)	(1.35–3.60)	(2.70–6.80)	(2.50–5.29)	(1.71–4.79)	(1.63–4.12)	(5.00–10.3)	(4.39–6.83)
95%	2.10	1.99	4.46	3.89	2.72	2.52	7.00	5.60
95% CI	(1.23–3.55)	(1.18–3.23)	(2.80–6.87)	(2.58–5.33)	(1.61–4.42)	(1.53–3.93)	(4.74–9.91)	(4.22–6.66)
**Child-B **^**b**^								
5%	2.76	2.60	4.22	3.73	4.21	3.72	7.73	6.20
95% CI	(1.62–4.64)	(1.56–4.11)	(2.56–6.69)	(2.39–5.38)	(2.56–6.68)	(2.39–5.37)	(5.28–10.8)	(4.65–7.52)
50%	2.24	2.13	4.27	3.75	3.99	3.54	7.05	5.63
95% CI	(1.31–3.79)	(1.27–3.44)	(2.65–6.66)	(2.46–5.20)	(2.45–6.30)	(2.28–5.01)	(4.77–9.98)	(4.25–6.74)
95%	1.89	1.80	4.25	3.71	3.91	3.45	6.56	5.21
95% CI	(1.10–3.21)	(1.07–2.96)	(2.67–6.56)	(2.47–4.99)	(2.42–6.12)	(2.25–4.78)	(4.42–9.37)	(3.96–6.15)
**Child-A **^**c**^								
5%	3.87	3.52	5.40	4.62	5.39	4.61	7.73	6.20
95% CI	(2.28–6.36)	(2.17–5.27)	(3.34–8.32)	(3.07–6.32)	(3.34–8.31)	(3.06–6.31)	(5.28–10.8)	(4.65–7.52)
50%	3.15	2.92	5.20	4.44	4.96	4.28	7.05	5.63
95% CI	(1.85–5.29)	(1.77–4.49)	(3.26–7.96)	(3.00–5.88)	(3.09–7.66)	(2.86–5.75)	(4.77–9.98)	(4.25–6.74)
95%	2.66	2.49	5.00	4.26	4.73	4.07	6.56	5.21
95% CI	(1.56–4.47)	(1.50–3.91)	(3.16–7.64)	(2.92–5.51)	(2.96–7.29)	(2.74–5.39)	(4.42–9.37)	(3.96–6.15)

P(ET)_avg_, extrathoracic particle penetration which is the thoracic particle fraction averaged across all activity levels weighted by daily ventilation; P(TB)_avg_, tracheobronchial particle penetration which is the respirable particle fraction averaged across all activity levels weighted by daily ventilation; 95% CI, ninety-five percent confidence intervals for inter-individual variability attributable to differences in particle penetration through the extrathoracic region; ^a^ Aerodynamic particle diameter in μm; ^b^ Scaling factor in Equations 2 and 3 equal to 1.26; ^c^ Scaling factor in Equations 2 and 3 equal to 1.0.

## Discussion

We calculated thoracic and respirable particle fractions for an adult male, adult female, and ten year-old child engaged in typical daily activities ranging from sleep to heavy exercise. Our estimates are intended to represent full-day ambient and/or non-ambient exposures while individuals are engaged in a variety of activities. This differs from the ACGIH and CEN criteria which are intended to represent a workplace setting [2,4]. Similarly, considering the need to provide protection for sensitive individuals who may breathe by mouth and/or oronasally, the EPA [8] selected the nominal cut-point of 10 μm as an indicator of the thoracic fraction consistent with ISO [26,27] recommendations for occupational or non-occupational environments. Our estimates show less penetration of coarse particulate matter into the thoracic and gas exchange regions of the respiratory tract than current criteria. For typical breathing habits (i.e., not oral breathing), we would predict less than 20% penetration of 10 μm particles into the thorax, whereas a 50% penetration of 10 μm is currently used in both occupational and non-occupational criteria [2,4,8,9]. Recognizing that there are differences in the sources and chemical composition between ambient fine (nominal mean d_a_ ≤ 2.5 μm) and larger coarse PM, our finding may, in part, explain why causal relationships are observed between morbidity and mortality with short and long-term exposure to fine PM but not larger coarse PM (see Chapter 2 in Ref [28]).

There are two primary reasons for the dramatic difference between our estimates and the current criteria. First, the ACGIH [4] criteria considered all inspired air to enter via the oral airway which increases the penetration through the ET airways. With the exception of a laboratory setting, however, few individuals breathe exclusively through the mouth. This would make the breathing habits other than oral breathing preferable for the purposes of estimating actual exposures. Second, the ACGIH criteria are intentionally conservative (Figure 1) as the committee chose to afford extra protection by over representing the true penetration of particles into the lower respiratory tract. In Figure 2, we predicted a 50% cut-point of 7.0 μm for the conditions considered by the ACGIH, namely, an orally breathing adult male engaged in light exercise. Additionally, our predicted upper bound 95^th^ percentile for 50% cut-points during oral breathing corrected for inhalability in Table 7 are ~10 μm. Thus, selection of 10 μm as having 50% penetration into the thorax was consistent with over representing the true penetration of particles into the lower respiratory tract of most individuals.

Route of breathing has a dramatic affect on particle delivery to the thoracic and respiratory regions since the deposition efficiency of the nasal passages greatly exceeds that of the oral pathway. Most subjects in the Niinimaa et al. [17] study, 87% (26 of 30), breathed through their nose until an activity level was reached when they switched to oronasal breathing. Thirteen percent (4 of 30) of the subjects, however, were oronasal breathers even at rest. These two subject groups are commonly referred to in the literature (e.g., see [12]) as “normal augmenters” and “mouth-breathers,” respectively. Becquemin et al. [18] and Bennett et al. [19] showed that children tend to have a greater fraction of oral breathing than adults at rest and during exercise. Route of breathing may also vary between races; Bennett et al. [20] found that African-Americans and females had a greater nasal contribution to breathing during exercise than Caucasians and males. The abrupt change in route of breathing occurring in normal augmenters has not been observed by others. The gradual augmenter breathing habit based on Bennett et al. [19,20] may be preferable to the normal augmenter in representing the general population. Chadha et al. [29] found that the majority (11 of 12) of patients with asthma or allergic rhinitis also breathe oronasally at rest. In healthy individuals, a small fraction (around 5%) may breathe solely through the mouth [21]. Our estimates for gradual augmenters provide particle penetration fractions most typical of healthy populations. Our estimates for mouth-breathers may be more appropriate for patients with mild upper respiratory disease.

The ICRP model [12] appears to underestimate the penetration of particles through the ET airways of children. A SF_t_ is applied in Equations 1-3 with the presumption that oral and nasal particle deposition increase with decreasing body size and increasing flow resistance.

For oral breathing on a mouthpiece, Bennett et al. [30] showed greater ET deposition in children than adults. This finding suggests that the application of a SF_t_ of 1.26 in Eq 1 is appropriate for laryngeal deposition. However, for nasal breathing, Becquemin et al. [22] and Bennett et al. [19] showed less nasal deposition in children than in adults. These two studies, not considered in the ICRP model [12], suggest that it may be inappropriate to apply a SF_t_ in Equations 2 and 3 for nasal deposition in children. Lower nasal deposition of particles in children than adults means greater penetration of particles into the lower respiratory tract of children than adults. Accordingly, we conducted simulations for the child with the SF_t_ in Equations 2 and 3 set equal to 1.0 in addition to the SF_t_ of 1.26 recommended by ICRP [12]. The estimated nasal ET deposition efficiency of 2 μm particles in the normal augmenter child during light exercise decreased from 68% to 48% when the SF_t_ was decreased from 1.26 to 1.0. For comparison, under the same level of activity, the estimated ET deposition efficiency was 57-58% in the adult male and female. Decreasing the nasal deposition efficiency of the child relative to the ICRP model [12] increased the particle size estimated to have 50% penetration into the thoracic and respiratory regions (see Tables 5, 6, 7 and Figures 4 and 5). These estimates of larger 50% cut points for child-A than adults appear consistent with studies in children that were not incorporated into the ICRP model [12].

With the exception of Table 7, the thoracic and respirable fractions that we present are the amount of particles entering a specified respiratory tract region relative to the amount of particles entering the respiratory tract. In effect, we assumed 100% inhalability across the range of particle sizes (0.5-20 μm) examined. We have opted on this convention since the inhalable fraction depends on factors not considered here such as wind speed and direction relative to the exposed individual. For recent reviews of the literature on particle inhalability, the reader is referred to Brown [31] and Millage et al. [32]. Adjusting our data for inhalability, shifts penetration curves to smaller particle sizes, but mainly only where there is a substantial contribution of oral breathing (see Table 7 vs. Table 5).

## Conclusion

Our analyses show that occupation and non-occupational criteria for thoracic and respirable fractions overestimate the size of particles entering these regions. As already noted, penetration fractions for workplace criteria were chosen to afford extra protection by over-representing the true penetration of particles into regions of the respiratory tract [4]. However, accepted definitions for thoracic and respirable fractions speak specifically to particles that penetrate into these regions. As such, current occupational and non-occupational criteria may misinform practitioners with regard to the actual size of particles expected to reach regions of the respiratory tract during typical behavior. For instance, the current criteria suggest that 10 μm particles (50%) penetrate into the thorax, thus, leaving the expectation that observed health effects may be modulated by their deposition in either the upper or lower airways. However, we predict that about 20% or less of these 10 μm particles would penetrate through the ET airways and into the lower respiratory tract. Our modifications to the ICRP model [12] related to breathing habit and nasal deposition in children reflect more recent data and provide consistent estimates of greater particle penetration into the thoracic and respiratory regions of children than adults. With those modifications, for median activities, we predict 50% cut-points for P(ET)_avg_ at ~3 μm in adults and ~5 μm in children. The predicted 50% cut-points for P(TB)_avg_ are slightly less than 3 μm in adults and slight greater than 4 μm in children. Our estimates of particle penetration into the thoracic and respiratory regions of the respiratory tract should be useful in the design of experimental studies and interpretation of PM health effects evidence.

## Endnotes

^a^More typically, the literature has defined this term in relation to the fraction of particles entering the gas-exchange region or the fraction penetrating through the tracheobronchial region, the ciliated airways, or conducting airways.

^b^For completeness, other groups such as the British Medical Research Council offered size-selective sampling recommendations prior to the ACGIH. For a historical perspective, the reader is referred to Lippmann [33].

^c^For accuracy it should be recognized that the sampler collection efficiency curves for EPA’s PM_10_ and ACGIH’s thoracic fraction are different. The criteria are similar for particles smaller than the 50% cut-point at 10 μm. However, the curves diverge at about 12 μm, with a dramatic drop in collection efficiency (dictated by policy considerations) for EPA’s PM_10_, and a more gradual decrease in collection efficiency for the ACGIH criterion.

^d^The MPPD model typically outputs estimates of regional deposition for the entire respiratory cycle. For the purposes of this project, the software output was modified by the developers to provide inspiratory deposition fractions for particles in the ET and TB regions. Designating the ET and TB regions as separate compartments, the deposition efficiency in the TB region (η_TB_) during inhalation was calculated from the MPPD output as DF_TB_ / (1-DF_ET_), where DF_TB_ and DF_ET_ are the deposition fractions of particles in the TB and ET region during inhalation, respectively. For more information about this model, the reader is referred to: http://www.ara.com/products/mppd_capabilities.htm.

## Abbreviations

ACGIH: American Conference of Governmental Industrial Hygienists; CEN: European Committee for Standardization; da: Aerodynamic diameter; DFTB: Particle deposition fraction in the TB region during inhalation; DFET: Particle deposition fractions in the ET region during inhalation; EPA: U.S. Environmental Protection Agency; ET: Extrathoracic; f: Breathing frequency; Fm: Fraction of breath passing through the mouth; FRC: Functional residual capacity; ICRP: International Commission on Radiological ProtectionISOInternational Organization for Standardization; MPPD: Multiple path particle dosimetry; NAAQS: National ambient air quality standard; ηTB: Particle deposition efficiency in the tracheobronchial region; η(ET)larynx: Extrathoracic particle deposition efficiency in the larynx; η(ET1)nose: Extrathoracic particle deposition efficiency in anterior nasal region; η(ET2)nose: Extrathoracic particle deposition efficiency in posterior nasal region; P(ET): Particle penetration past the larynx and the thoracic fraction; P(TB): Particle penetration through the ciliated airways and the respirable fraction; PM: Particulate matter; PM2.5: Particles with a nominal mean aerodynamic diameter ≤ 2.5 μm; PM10: Indicator for thoracic coarse particles; Qnose: Inspiratory flow through the nose; Qtotal: Total inspiratory flow rate; SFt: Scaling factor, ratio of trachea diameter in adult reference male to that of subject; t: Time spent engaged in specific activity; TB: Tracheobronchial; TSP: Total suspended particulate; URT: Upper respiratory tract volume; Vdaily: Total volume inspired in 24 hours; VT: Tidal volume.

## Competing interests

The authors have no competing interest.

## Authors’ contributions

JB conceived the project, coordinated and drafted the manuscript. TG contributed to defining the project’s scope and drafting the manuscript. OP modified software for the purposes of this project to provide inspiratory deposition fractions for particles and participated in the interpretation of data. BA contributed to the methodology, participated in software development and drafting of the manuscript. All authors read and approved the final manuscript.

## Supplementary Material

Additional file 1 Comparison of respiratory particle fractions predicted by the MPPD and ICRP [12] models. In general, the ICRP [12] model predicts less particle penetration into the respiratory region than the MPPD model. Click here for file
