# Supplementary material for: Thoracic and respirable particle definitions for human health risk assessment
Source: Part Fibre Toxicol. 2013 Apr 10;10:12. doi: 10.1186/1743-8977-10-12 (PMC3640939; doi:10.1186/1743-8977-10-12)
Supplement: Additional file 1 — Comparison of respiratory particle fractions predicted by the MPPD and ICRP [12] models. In general, the ICRP [12] model predicts less particle penetration into the respiratory region than the MPPD model. [file 1743-8977-10-12-S1.pdf]

APPENDIX

Within this appendix we compare the respiratory particle fraction estimated in our paper using the MPPD model to estimates obtained using the ICRP [10] human respiratory tract model. As discussed in our paper, the ICRP human respiratory tract model [10] was used to estimate particle penetration through the ET airways. Particles that do not deposit in the ET region of the respiratory tract enter the lungs; however, their regional deposition within the lungs cannot be precisely measured. In ascertaining regional lung deposition, there are uncertainties due to slow particle clearance from the TB airways and the penetration of even shallowly inhaled aerosol boluses into the alveolar region. Due to these uncertainties, we opted to utilize the publicly available multiple path particle dosimetry (MPPD; ver 2.1, © 2009) model to estimate penetration through the TB airways. Nonetheless, the use of the ICRP [10] model for TB deposition may be viewed as preferable since it is based on human experimental data, whereas the MPPD model is deterministic based on theoretical deposition in a series of tubes.

By convention, penetration into the respiratory region is the amount of inhaled material minus the amount deposited in the ET and TB airways during inhalation. Beyond the calculations of ET deposition (see Equations 1-4 of our paper), equations necessary to calculate deposition in the TB airways are provided in Tables 12 and 13 of the ICRP [10] model. Additional dead space volumes and scaling factors necessary for these calculations were taken from Table 15 of ICRP [10]. All other necessary parameters are consistent with those already provided in our paper. We did not estimate diffusive deposition in the ET airways since aerodynamic losses dominate in this region even for the smallest particles we evaluated. However, it was necessary to consider diffusive deposition in the TB airways since

thermodynamic losses generally exceeded aerodynamic losses in these airways for particles <1 μm inhaled during activities of sleep and sitting.

Figure 1-A illustrates penetration through the TB airways, P(TB), into the respiratory region. In this figure, for particles >1.7 μm, the ICRP model predicts less P(TB) than the MPPD model. Across all combinations of breathing conditions, sex, and age, η_{TB} predicted by the ICRP model was lower than predicted by the MPPD model for small particles <1 μm. However, at between 1 and 3 μm and for larger particles, the η_{TB} predicted by the ICRP model exceeded that predicted by the MPPD model. The ICRP's greater η_{TB} lead to lower P(TB) than predicted by the MPPD model except in normal augmenters (see Table A-1). In normal augmenters, the particle losses in the nasal airways were extensive enough so as to have minimal differences in the predicted P(TB) between the models.

TABLE A-1. Comparison of respirable particle fraction, $P(TB)_{avg}$, predicted using ICRP and MPPD models. Data are relative to particles entering the respiratory tract.

V_{daily} (%-tile)	Normal Augmenter[a]		Mouth-breather[a]		Gradual Augmenter[a]		Oral only[a]	
	ICRP	MPPD	ICRP	MPPD	ICRP	MPPD	ICRP	MPPD
Male								
5%	2.72[b]	2.74	4.10	4.32	3.19	3.25	6.02	6.67
50%	2.28	2.30	3.84	4.30	2.91	3.05	5.22	6.11
95%	2.01	2.03	3.74	4.31	2.75	2.90	4.87	5.89
Female								
5%	2.69	2.71	3.61	3.78	2.82	2.86	5.91	6.50
50%	2.24	2.27	3.52	3.87	2.60	2.68	5.13	5.96
95%	1.98	1.99	3.50	3.96	2.45	2.54	4.82	5.77
Child-B[c]								
5%	2.60	2.61	3.68	3.78	4.68	3.78	5.95	6.46
50%	2.13	2.13	3.54	3.81	3.37	3.59	5.18	5.81
95%	1.81	1.81	3.38	3.76	3.19	3.49	4.64	5.33
Child-A[d]								
5%	3.49	3.56	4.51	4.72	4.50	4.72	5.95	6.46
50%	2.87	2.94	4.14	4.53	4.01	4.36	5.18	5.81
95%	2.44	2.50	3.85	4.34	3.70	4.14	4.64	5.33

[a]Data are daily averages, all activity levels weighted by daily ventilation; [b]Aerodynamic particle diameter in µm; [c]Scaling factor in Equations 2 and 3 equal to 1.26; [d]Scaling factor in Equations 2 and 3 equal to 1.0.

FIGURE A-1

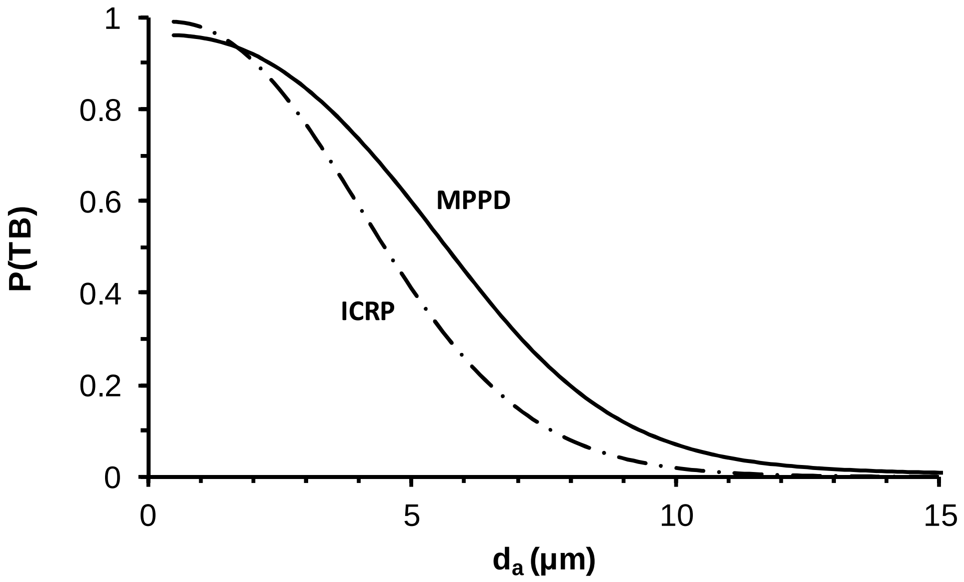

FIGURE A-1. Comparison of respirable particle fraction predicted using ICRP and MPPD models. Data are for an orally breathing adult male engaged in light exercise. Penetration through the tracheobronchial airways is relative to particles entering the respiratory tract.
